# Auricular acupuncture for chemically dependent pregnant women: a randomized controlled trial of the NADA protocol

**DOI:** 10.1186/1747-597X-7-48

**Published:** 2012-12-23

**Authors:** Patricia A Janssen, Louise C Demorest, Anne Kelly, Paul Thiessen, Ron Abrahams

**Affiliations:** 1 School of Population and Public Health, Child and Family Research Institute, University of British Columbia, 2206 East Mall, Vancouver, BC, Canada V6T-1Z3; 2 Toronto Central Local Health Integration Network, Toronto, ON, Canada; 3 Dept. of Pediatrics, University of British Columbia, British Columbia, Canada; 4 Dept of Medicine, University of British Columbia, British Columbia, Canada

**Keywords:** Acupuncture, Addiction, Pregnancy, Substance abuse, Neonatal abstinence syndrome

## Abstract

**Background:**

The prevalence of maternal drug use during pregnancy in North America has been estimated to be as high as 6-10%. The consequences for the newborn include increased risk for perinatal mortality and ongoing physical, neurobehavioral, and psychosocial problems. Methadone is frequently used to wean women off street drugs but is implicated as a cause of adverse fetal/neonatal outcomes itself. The purpose of our study was to test the ability of maternal acupuncture treatment among mothers who use illicit drugs to reduce the frequency and severity of withdrawal symptoms among their newborns.

**Methods:**

We randomly assigned chemically dependent pregnant women at BC Women’s Hospital in Vancouver, British Columbia to daily acupuncture treatments versus usual care. By necessity, neither our participants nor acupuncturists were blinded as to treatment allocation. Our primary outcome was days of neonatal morphine treatment for symptoms of neonatal withdrawal. Secondary neonatal outcomes included admission to a neonatal ICU and transfer to foster care.

**Results:**

We randomized 50 women to acupuncture and 39 to standard care. When analyzed by randomized groups, we did not find benefit of acupuncture; the average length of treatment with morphine for newborns in the acupuncture group was 2.7 (6.3) compared to 2.8 (7.0) in the control group. Among newborns of women who were compliant with the acupuncture regime, we observed a reduction of 2.1 and 1.5 days in length of treatment for neonatal abstinence syndrome compared to the non-compliant and control groups, respectively. These differences were not statistically significant.

**Conclusions:**

Acupuncture may be a safe and feasible treatment to assist mothers to reduce their dosage of methadone. Our results should encourage ongoing studies to test the ability of acupuncture to mitigate the severity of neonatal abstinence syndrome among their newborns.

**Clinical Trial Registration:**

http://www.clinicaltrials.gov registry: W05-0041

## Background

The prevalence of maternal drug use during pregnancy in Canada [1] and the US [2] has been estimated to be as high as 6-10%. The consequences for the newborn are severe, and include increased risk for perinatal mortality and morbidity, as well as ongoing physical, neurobehavioral, and psychosocial problems [3-7]. Between 60-90% of infants exposed to illicit drugs in utero will exhibit clinical symptoms of withdrawal, particularly if opiates are among the drugs used [8-10].

Methadone treatment is currently the recommended approach to opiate addiction during pregnancy [11]. The purpose of this treatment is to alleviate the symptoms of drug withdrawal in order to prevent use of street drugs. It is widely believed that intrauterine death or fetal compromise is due to the “bingeing” or drastic fluctuations in serum levels of opiates or cocaine associated with “street” use [12]. Methadone substantially minimizes the peak and trough in maternal serum opioid levels that typically occur with repeat use of short-acting opioids such as heroin, thereby reducing the harm that the fetus encounters as a result of repeated intoxication and withdrawal [13]. Methadone use during pregnancy has been associated with improved prenatal care, [14] longer gestation, [15] higher birthweight, [16] and increased rates of infants discharged home in the care of their mothers [17]. In hospital, the dosage of methadone is tailored to the mother’s tolerance of symptoms and is titrated over a 24-hour period to avoid fluctuations in levels of methadone in the fetal circulation. Unfortunately, if symptoms are not well controlled with methadone use, or perhaps even if they are, women frequently continue to use other drugs [18]. Methadone itself is highly addictive and is implicated as a cause of adverse fetal/neonatal sequelae [19,20]. Methadone-exposed babies have demonstrated reduced birth weight and head circumference, prematurity, [21] and rates of neonatal withdrawal syndrome ranging from 46% [22] to 97% [12,23]. A dose–response relationship between methadone and neonatal abstinence syndrome (NAS) has been reported in a large series of 618 women [24] but a meta-analysis by the same author [23] and others [25] did not confirm these findings. A recent prospective study by this group concluded that neonates exposed to methadone doses ≥ 80 mg required higher cumulative doses of morphine treatment for NAS but attributed this to concomitant use of other drugs [26]. Increased rates of congenital anomalies have been reported in non-randomized studies, [12,24,27] but these have not yet been evaluated in randomized designs [28] capable of controlling for concomitant consumption of other substances.

While the physiological mechanisms underlying acupuncture have yet to be unambiguously identified, the balance of evidence favours quantitative differences in electrodermal properties between acupuncture points and surrounding skin [29,30]. There is also emerging evidence of correlations between connective tissue anatomy, microcirculatory blood flow, and acupuncture needling points [31]. Acupuncture in the context of addiction is still relatively new [32-35]. Acupuncture points are cutaneous areas containing relatively large concentrations of free nerve endings [36]. The acupuncture stimulus is transmitted to the spinal cord by afferent peripheral nerves [34]. Acupuncture is believed to stimulate the release of endorphins [37] from the pituitary gland and the hypothalamus. Endorphins are neurotransmitters involved in pain inhibition. They are 10–100 times more potent than morphine and may circulate for several hours. Levels of endorphins have been shown to increase after acupuncture in animal models [38,39]. Alcohol and opioids may preferentially bind to endorphin receptors and thereby displace endogenous endorphins [40,41]. Over time the production and action of the natural endorphins is inhibited. Craving during withdrawal from illicit opioids may result from a deficiency in endogenous opioids as well as from other neurochemical defects associated with drug abuse [40].

The role of acupuncture in the treatment of addiction was discovered serendipitously [42] and subsequently refined at the Lincoln Recovery Center in Bronx, New York where the National Acupuncture Detoxification Association (NADA) protocol was developed [43]. A randomized controlled trial of NADA auricular acupuncture utilizing analysis of urine screens showed that acupuncture significantly reduced cocaine use compared with each of two control conditions [32].

There have been no studies to date specifically addressing the use of acupuncture for treatment of substance abuse in pregnancy. The purpose of this study is to determine the efficacy of daily maternal acupuncture treatments in reducing the frequency and severity of (NAS) among infants born to substance-using women.

## Methods

We conducted a randomized controlled trial of methadone maintenance (standard treatment) to chemically dependent hospitalized pregnant women vs. methadone combined with the offer of daily acupuncture treatments. Our primary outcome was the number of days that neonates were treated with morphine for neonatal withdrawal syndrome. We received ethical approval from the University of British Columbia Clinical Ethics Research Board and the BC Women’s Hospital Research Review Committee.

### Setting

We conducted our study at BC Women’s Hospital in Vancouver, British Columbia between July 15, 2005-April 30, 2008. Chemically dependent women living in Vancouver and surrounding suburbs are referred to the BC Women’s Chemical Dependency Unit by their primary caregiver as soon as they present for prenatal care, usually in the second trimester. They are admitted to the chemical dependency unit on a voluntary basis. They are offered a methadone maintenance program or support to withdraw from methadone and other illicit drugs. After an initial stay of approximately two months, they are discharged then readmitted approximately two weeks prior to their due date. After the birth, mothers and their newborns are discharged together when the baby is stable, gaining weight, and does not require treatment for symptoms of NAS. The unit is built on an empowerment model in which women have access to a variety of “healing” activities such as yoga, gardening, therapeutic touch, peer support groups, arts and crafts, group walks and massage therapy. Residents participate in their own discharge planning meetings. Sessions with alcohol and drug support counsellors are available on the unit. Urine testing is not done. Babies room in with their mothers unless neonatal intensive care is required. All women admitted to the Chemical Dependency Unit at BC Women’s Hospital were offered participation in the current study.

### Sample

Women admitted to the chemical dependency unit at BC Women’s Hospital, Vancouver, B.C. were considered to be eligible for inclusion. Exclusion criteria consisted of inability to read or write English, having a pacemaker or other electrical implant, having a bleeding disorder, or a condition putting someone at particular risk for infection, including for example, damaged heart valves, diabetes requiring insulin, immunosuppressive drug therapy or open wounds.

### Outcome measures

Number of days of treatment of the newborn with morphine was chosen as the primary outcome because it is a clinical measure of the time required for the newborn to complete withdrawal from opiates. In this unit, morphine is prescribed for the neonate by pediatricians if there is a constellation of symptoms unresponsive to environmental control including: 1) convulsions, 2) inconsolability or crying continuously for 3 hours, 3) persistent tremors or jitteriness when undisturbed, 4) continuous central nervous system irritability including hyperactive Moro reflex, tremors, jitteriness, increased muscle tone and unprovoked muscle jerks, 5) persistent vomiting or projectile vomiting over a 12 hour period, or 6) explosive diarrhea for 2–3 consecutive episodes [44]. Additional clinical signs such as tachycardia, tachypnea, watery stools, fever, or weight loss > 10% may justify use of morphine after consideration of differential diagnoses. Morphine 1 mg/ml is started at a rate of 0.03 mg/kg/dose every 3 hours. The dose is reviewed daily and titrated based on daily weights and ongoing symptoms.

Secondary neonatal outcomes include gestational age at birth, Apgar scores, days to regain birth weight, rates of admission to a neonatal intensive care nursery, withdrawal symptoms and rates of transfer of the infant to foster care. Infants experiencing NAS have been shown to require significantly longer time to regain their birthweight [45]. Neonatal outcomes were ascertained from the hospital chart by a research assistant blinded to study allocation. Withdrawal symptoms are routinely documented by nurses using a modified version of the Finnegan Scale [46]. Nurses were not formally blinded to study allocation. The Finnegan scale has been widely used in studies of NAS [47,48] and has been shown to be a valid standard against which pharmacologic treatment can be titrated [49,50]. The original 22-item-scale instrument consists of variables such as sleep duration after feeding, mottling, and nasal stuffiness. We report on a subset of more objective items that are documented daily on the unit including high pitched cry, inconsolable crying, tremors, muscle tone, sucking and swallowing, vomiting and diarrhea.

### Study protocol

Women were recruited on the unit by a trial coordinator. After obtaining written informed consent a sequentially numbered opaque envelope was opened to reveal the study allocation by the study research assistant. Random allocation to study arm was undertaken using statistical software, SPSS version 18.

We used the National Acupuncture Detoxification Association (NADA) five-point auricular acupuncture protocol for treating symptoms of drug withdrawal [43]. The protocol consists of inserting five stainless steel acupuncture needles in both ears at points known as Sympathetic, Shen men, Liver, Kidney, and Lung. This point combination is believed to be specific for substance abuse. The acupuncturist swabbed the ears with alcohol and inserted sterile, disposable needles. Following the 45 minute treatment, participants removed the needles and placed them in protective sharps boxes in order to minimize risk of needlestick injury to the acupuncturist. All needles were counted to ensure that all had been retrieved and disposed of. A sham acupuncture procedure was not used. Chinese traditional medicine does not include the concept of a placebo [51]. Those who argue that auricular acupuncture stimulates the vagus nerve, which innervates the ear concha, state that needles placed anywhere in the concha should produce the same effects [52]. Studies utilizing sham procedures have failed to show a difference between the control and active experimental conditions [51,53].

Women participating in the treatment group of our study were given access to a quiet room furnished with comfortable reclining chairs. The acupuncturist spent approximately 30 minutes with them each day.

Physicians prescribing morphine to newborns were blinded as to treatment arm. Assignment to trial arm was not written in the chart. Women received acupuncture treatment at mid-day when pediatricians were not usually on the unit and in a room with the door shut. Mothers were asked not to tell physicians if they were receiving acupuncture.

### Sample size

We planned to have 80% power to detect a 30% reduction of days of neonatal morphine treatment, from 11.75 (5.2) to 8.25 days (5.2) with 37 subjects per treatment arm. The baseline rate of 11.75 days was derived from a pilot study of this population at BC Women’s by one of the authors (RA) [54].

### Data analysis

Data analysis was by intention to treat. Outcomes of participants were analyzed within the trial arm that they were randomly assigned to. Socio-demographic characteristics assessed at baseline included age, marital status, ethnicity, income, parity, housing (stable vs. transient), smoking status and education. Pregnancy-related characteristics assessed included pre-pregnant weight and weight gain, month of entry to prenatal care, and self-reported substance use. Tests of normality (Kolmogorov-Smirnov) were applied to continuous variables. Normally distributed continuous variables were compared between groups using the *t*-test. Non- normally distributed variables were compared using the Mann–Whitney *U* test for two groups and the Kruskal-Wallis test for three groups. Discrete variables were compared between groups using the chi-square statistic when expected cell counts were greater than five; otherwise the Fisher’s exact test was reported. Statistical analysis was undertaken using SPSS, version 18.

## Results

**Figure 1:**
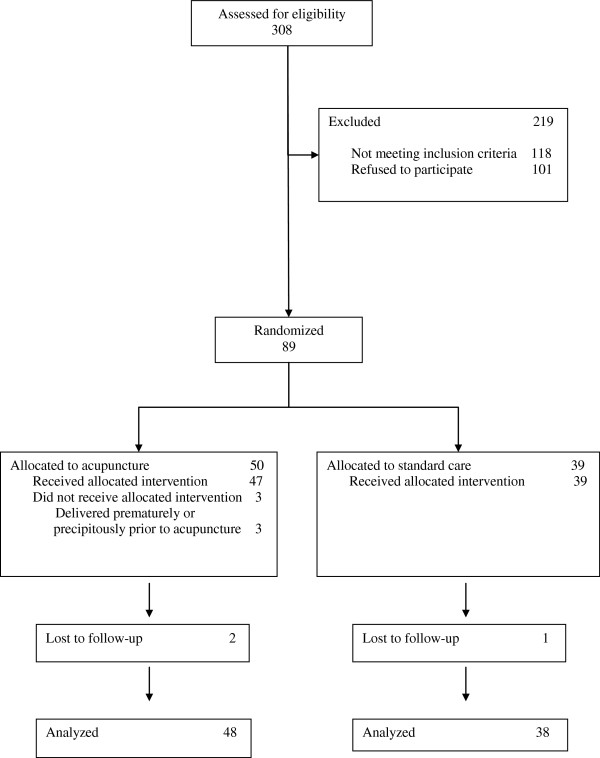
Flow chart of Participant Eligibility, Recruitment, and Compliance.

Among 190 eligible women approached to participate in the study, 89 agreed to participate (Figure 1). Three women in the acupuncture group delivered prematurely or precipitously prior to receiving a treatment. Outcomes could not be ascertained for 2 women in the acupuncture group and one in the control group because they delivered outside of BC Women’s Hospital at an unknown location. Forty-seven women received acupuncture treatments and 39 received standard ward care. Study groups were comparable with respect to demographic status including age, presence of support persons, education, and sources of income (Table 1). Groups did not differ with respect to substance use in previous pregnancies, retention of custody of previously born children, and use of alcohol, tobacco, crack, cocaine, crystal meth, heroin or other opioids, benzodiazepines, or ecstasy (Table 2). Women in each study arm did not differ with respect to pre-pregnant weight, height, weight gain during the pregnancy, or gestational age at entry to prenatal care (Table 3). The average length of stay on the unit was similar in the acupuncture and control groups. Dosage of methadone on admission to hospital was higher on average by 10 mg in the acupuncture group. Mode of delivery and rates of breastfeeding did not differ between groups. Analyzed by intention to treat, there were no differences in days of morphine treatment between treatment groups. Similarly, groups did not differ with respect to newborn Apgar scores at one or five minutes, admission to neonatal intensive care unit (ICU), days to regain birthweight, apprehension of the baby to foster care, or symptoms of neonatal abstinence (Table 4).

**Table 1:** Sociodemographic profile of study participants

	**Acupuncture n= 50**	**Control n= 39**	**P-value**
Age (years), mean (sd) ^a^	28.2 (5.6)	29 (5.9)	0.44
Support, n (%) ^b^			
Lone Parent	19 (38.0)	13 (33.3)	0.65
Birth Father involved	23 (46.0)	21 (53.8)	0.46
Other Partner	5 (10.0)	5 (12.8)	0.68
Family	21 (42.0)	14 (35.9)	0.56
Education, n (%)^b^			
Elementary	1 (2.0)	0 (0.0)	0.83
Some high school	25 (51.0)	22 (61.1)	
High School Diploma	9 (18.4)	5 (13.9)	
Some post-secondary	7 (14.3)	5 (13.9)	
Diploma for Trade School	5 (10.2)	4 (11.1)	
Some university	1 (2.0)	0	
University Degree	1 (2.0)	0	
Ethnicity, n (%)^b^			
Caucasian	36 (72.0)	23 (59.0)	0.23
First Nations	10 (20.0)	14 (35.9)	
Chinese	2 (4.0)	0	
South Asian	2 (4.0)	1 (2.6)	
African Canadian	0	1 (2.6)	
Income, n (%)^b^			
Employed	3 (6.0)	1 (2.6)	0.98
Social Assistance	31 (62.0)	26 (66.7)	
Reliant on partner	3 (6.0)	1 (2.6)	
No source of income	3 (6.0)	3 (7.7)	
Source unknown	2 (4.0)	2 (5.1)	
Disability	3 (6.0)	3 (7.7)	
Sex trade	3 (6.0)	2 (5.1)	
Incarcerated	2 (4.0)	1 (2.6)	

^a ^Mann–Whitney *U *test.

^b ^Chi square test, 2df, except education (6df), ethnicity, (4df), income (8df).

**Table 2:** Substance use profile of study participants on admission to hospital

	**Acupuncture n= 50**	**Control n= 39**	**P-value**
Smoking, n (%) ^a^			
Current	44 (88.0)	37 (94.9)	0.50
Former	4 (8.0)	1 (2.6)	
Never	2 (4.0)	1 (2.6)	
Cigarettes per day, mean (sd) ^b^	13.0 (8.0)	12.4 (5.6)	0.83
Alcohol, n (%)^a^			
Daily	2 (4.1)	3 (7.7)	0.52
Weekly	0	1 (2.0)	
Sporadically during month	8 (16.3)	8 (20.5)	
None	39 (79.8)	27 (69.2)	
Heroin, n (%)^a^			
Daily	19 (38.0)	14 (35.9)	0.63
Weekly	2 (4.0)	0	
Sporadically	5 (10,0)	4 (10.3)	
None	24 (48.0)	21 (53.8)	
Methadone, n (%)^a^			
Daily	21 (42.9)	14 (35.9)	0.61
Weekly	14 (28.8)	15 (38.5)	
None	14 (28.6)	10 (25.8)	
Other Opioid, n (%)^a^			
Daily	2 (4.0)	3 (7.7)	0.74
Sporadically	1 (2.0)	1 (2.8)	
None	47 (94.0)	35 (89.7)	
Cocaine, n (%)^a^			
Daily	12 (24.0)	8 (20.5)	0.86
Weekly	2 (4.0)	2 (5.1)	
Sporadically	6 (12.0)	3 (7.7)	
None	30 (60.0)	26 (66.7)	
Crack, n (%)^a^			
Daily	23 (46.0)	15 (38.5)	0.78
Weekly	1 (2.0)	2 (5.1)	
Sporadically	8 (16.0)	6 (15.4)	
None	18 (36.0)	16 (41.0)	
Cannabis, n (%)^a^			
Daily	2 (4.0)	7 (17.9)	0.08
Weekly	3 (6.0)	0	
Sporadically	8 (16.0)	5 (12.8)	
None	37 (74.0)	27 (69.2)	
Crystal Meth, n (%)^a^			
Daily	4 (8.0)	4 (10.3)	0.96
Weekly	2 (4.0)	1 (2.6)	
Sporadically	6 (12.0)	4 (10.3)	
None	38 (76.0)	30 (76.9)	
Benzodiazepine, n (%)^a^			
Daily	0	3(7.7)	0.14
Sporadically	3 (6.0)	2 (5.1)	
None	47 (94.0)	34 (87.2)	
Ecstasy, n (%)^a^			
Daily	0	1 (2.8)	0.52
Sporadically	3 (6.0)	2 (5.1)	
None	47 (94.0)	36 (82.3)	
Antidepressant, n (%)^a^			
Daily	4 (8.2)	2 (5.1)	0.85
Sporadically	1 (2.0)	1 (2.5)	
None	44 (89.8)	36 (92.3)	
Methadone dose (mg), mean (sd) ^b^ (n=36,28)			
During pregnancy	52.9 (49.7)	49.2 (47.2)	0.85
Admission to hospital	48.8 (50.6)	40.0 (39.0)	0.62
At delivery	58.1 (53.6)	48.4 (50.4)	0.45
Gestational age when started (wks)	14.8 (12.3)	13.5 (13.2)	0.42
Non-opioid users, n (%)^a^	10 (20)	11 (24.2)	0.32
Use of substances when pregnant with other children, n (%)^a^	22/38 (57.9)	19/31 (61.3)	0.78
Retained custody of at least one child, n (%)^a^	19/36 (52.8)	16/29 (55.2)	0.85
Treatment Goal, n (%)^a^			
Abstinence	36 (72.0)	30 (76.9)	0.57
Decrease methadone	13 (26.0)	7 (17.9)	
Decrease other drug	0	1 (2.6)	
No goal	1 (2.0)	1 (2.6)	

^a ^Chi square test, 2df (smoking, methadone, other opioid, benzodiazepine, ecstasy, antidepressant) 3df (all other categorical variables).

^b ^Mann–Whitney *U* test.

**Table 3:** Pregnancy-related characteristics of participants

	**Acupuncture n= 50**	**Control n= 39**	**P-value**
Gravidity, mean (sd) ^a^	3.9 (2.2)	3.8 (2.2)	0.75
Parity, n (%) ^b^			
0	14 (28.0)	10 (25.6)	0.97
1	14 (28.0)	11 (28.2)	
2+	22 (44.0)	18 (46.2)	
Pre-Pregnancy Weight (kg), mean (sd)^a^	61.1 (13.7)	64.8 (17.7)	0.39
Height (cm), mean (sd) ^a^	164.2 (7.9)	164.1 (5.8)	0.81
Weight Gain (kg), mean (sd) ^a^	15.7 (14.0)	13.9 (12.4)	077
Gestational age at hospital admission (weeks), mean (sd) ^a^	19.1 (9.4)	19.2 (9.5)	0.90
Prenatal Classes, n (%)^b^			
Yes	4 (8.3)	4 (10.8)	
No	33 (68.9)	27 (73.0)	
In previous pregnancy	11 (22.9)	6 (16.2)	0.72
Psychiatric Diagnosis, n (%)^b^			
None	23 (46.0)	25 (64.1)	0.07
Depressed	9 (18.0)	7 (17.9)	
Bipolar	10 (20.0)	1 (2.6)	
Anxiety Disorder	5 (10.0)	4 (10.3)	
Psychosis	2 (4.0)	0	
Borderline personality	1 (2.0)	0	
Days AWHOL, mean, (sd) ^a^	15 (30.8)	10 (25.6)	0.89

^a ^Mann –Whitney *U *test.

^b ^Chi square test, 2df.

**Table 4:** Maternal and newborn outcomes

	**Acupuncture n= 48**	**Control n= 38**	**P-value**
Breast Feeding, n (%) ^a^			
Exclusive	12 (25.5)	7 (20.0)	
Combined	17 (36.2)	15 (42.9)	
Formula	18 (38.3)	13 (37.1)	0.78
Missing	1	3	
Mode of Delivery, n (%)^a^			
Spontaneous Vaginal	30 (62.5)	25 (65.9)	
Assisted Vaginal	4 (8.3)	2 (5.3)	
Cesarean	14 (29.2)	11 (28.9)	0.85
Gestational Age at Delivery (weeks), mean (sd) ^b^	38.0 (2.8)	38.0 (2.6)	0.37
Days in Hospital, mean (sd) ^b^	28.7 (23.9)	29.3 (25.6)	0.39
Apgar Score < 7 @ 1 minute, n (%) ^c^	10 (20.4)	8 (22.2)	1.00
Apgar Score < 7 @ 5 minute, n (%)^c^	1 (2.0)	3 (8.3)	0.31
Missing	1	3	
Birth Weight, mean (sd)^b^	2985.7 (594.7)	3074.9 (557.1)	0.26
Birth Length, mean (sd) ^b^	49.12 (5.1)	48.8 (4.3)	0.47
Head Circumference, mean (sd) ^b^	33.3 (1.6)	33.9 (1.90)	0.22
Days to regain birth weight, mean (sd)^b^	11.2 (4.7)	10.6 (3.9)	0.57
Days of treatment with morphine, mean (sd) ^b^	2.7 (6.3)	2.8 (7.0)	0.97
Admitted to NICU, n (%)^c^	19 (38.0)	11 (28.2)	0.37
Mother lost custody, n (%)^c^	18 (36.7)	16 (44.4)	0.51
Symptoms of Neonatal Abstinence			
Syndrome (days), mean (sd) ^b^			
High pitched cry	3.5 (5.0)	3.4 (6.1)	0.46
Crying inconsolably	0.3 (0.9)	0.4 (1.1)	0.94
Tremors/jitteriness when disturbed	8.6 (5.5)	8.7 (7.2)	0.58
Tremors/jitteriness when undisturbed	1.8 (3.0)	1.5 (2.8)	0.68
Abnormal muscle tone	5.0 (6.3)	5.7 (8.7)	0.61
Disorganized sucking/swallowing	2.2 (4.5)	2.6 (5.8)	0.57
Weak or absent suck	1.9 (3.8)	2.4 (5.3)	0.75
Vomiting	0.2 (1.0)	0.3 (1.0)	0.69
Loose, watery, or explosive stools	1.5 (3.5)	1.8 (3.0)	0.29

^a ^Chi square test , 2df.

^b ^Mann–Whitney *U *test.

^c ^Fisher’s Exact Test.

Compliance with the acupuncture regime varied greatly among participants, as did time spent on the hospital unit. When we compared compliant participants, defined as those who received the highest quartile of acupuncture treatments, (nine or more treatments), (n=13) with those who did not (n=27) in the acupuncture group, and controls (n=32) in a post hoc analysis, those in the compliant group were taking higher doses of methadone at delivery (Table 5). The reduction in use of methadone from first admission to hospital to delivery was larger in the acupuncture-compliant group compared to the acupuncture non-compliant group and the control group. These findings would suggest that women who require higher doses of methadone were more compliant with treatment, perhaps because they believed it to be helpful. On this basis we undertook an additional “as treated” analysis, comparing women who had nine or more acupuncture treatments with those who were non-compliant (less than nine) and control subjects. We further restricted this analysis to women who had some ingestion of opioid, as there were fourteen women in the study who ingested only crack or cocaine and two who were exposed only to crystal meth.

**Table 5:** Maternal and newborn outcomes in an “as treated” analysis among mothers exposed to opiates

	**Acupuncture compliant n= 13**	**Acupuncture non-compliant n=27**	**Control n=32**	**P-value**
Maternal methadone dose at delivery (mg), mean (sd) ^a^	75.2 (63.7)	67.0 (45.5)	64.5 (48.3)	0.86
Change in methadone dose from admission- until delivery, (mg), mean (sd)^a^	−15.9 (37.7)	−8.9 (34.8)	−11.6 (25.7)	0.85
Days of newborn treatment with morphine, mean (sd) ^a^	1.9 (4.5)	4.0 (7.8)	3.5 (7.7)	0.71
Apgar Score < 7 @ 1 minute, n (%) ^b^	2 (15.4)	7 (26.9)	6 (20.7)	0.80
Apgar Score < 7 @ 5 minute, n (%) ^b^	1 (7.7)	0	2(6.9)	0.41
Missing	0	1	3	
Admitted to NICU, n (%) ^b^	4 (30.8)	12 (44.4)	9 (28.1)	0.44
Mother lost custody, n (%) ^b^	5 (38.5)	7 (26.9)	12 (40/0)	0.54
Missing	0	1	2	
Symptoms of Neonatal Abstinence Syndrome (days), mean (sd) ^a^				
High pitched cry ^c^	2.1 (3.5)	4.0 (5.4)	3.4 (6.1)	0.83
Crying inconsolably	0.3 (1.0)	0.3 (1.0)	0.4 (1.1)	0.52
Tremors/jitteriness when disturbed	5.7 (2.5)	9.5 (5.9)	8.7 (7.2)	0.28
Tremors/jitteriness when undisturbed	0.4 (1.0)	2.3 (3.4)	1.5 (2.8)	0.24
Abnormal muscle tone	3.1 (4.0)	5.7 (6.9)	5.7 (8.7)	0.83
Disorganized sucking/swallowing	1.8 (4.4)	2.3 (4.6)	2.6 (5.8)	0.89
Weak or absent suck	1.3 (2.9)	2.1 (4.1)	2.4 (5.3)	0.98
Vomiting	0.1 (0.3)	0.3 (1.0)	0.3 (1.0)	0.86
Loose, watery, or explosive stools	0.6 (1.2)	1.9 (4.0)	1.8 (3.0)	0.21

^a ^ANOVA, Methadone dose at delivery, Change in methadone df 2, 48; Days of newborn treatment with morphine df 2, 64.

^b ^Fisher’s exact test.

^c ^Kruskall-Wallis test, 2df.

In this analysis newborns of women in the acupuncture-compliant group experienced a reduction of 2.1 and 1.5 days in length of treatment for neonatal abstinence syndrome compared to the non-compliant and control groups, respectively. These differences were not statistically significant (Table 5). With the exception of inconsolable crying, their newborns exhibited symptoms of NAS for fewer days. These differences were not statistically significantly different.

## Discussion

We report that the offer of acupuncture to hospitalized chemically dependent women in a randomized design is not associated with improved perinatal outcomes. In the current study, only 28% of women in our acupuncture arm were protocol-compliant. Women in our study who were compliant were found to be receiving higher doses of methadone on admission to hospital and reduced their dosage of methadone to a greater degree than either the non-compliant women in the acupuncture-group or the control group. This would suggest that women who would be expected to suffer most from withdrawal symptoms remained compliant because they believed that the acupuncture was helping them. Indeed they were able to tolerate larger reductions in their methadone dose prior to delivery and their babies required almost two fewer days of morphine treatment. As well, the babies in this small group were documented by nurses using a standardized symptom log to have had symptoms of neonatal withdrawal for shorter periods of time.

Factors promoting compliance have been understudied in women who are addicted to illicit drugs. Women in our study who were non-compliant most often told the acupuncturist that they were “too busy” to receive a treatment, although there was no apparent reason to be busy on the hospital ward. In a previous study of acupuncture in a non-pregnant population, the authors demonstrated a reduction in use of illicit drugs among participants who came consistently for acupuncture, but only 15 percent of attendees to the clinic remained compliant [34].

Non-compliance further limits our study by reducing the number of exposed subjects in our study arm and thus our power to detect differences. The non-statistically significant reduction in duration of morphine treatment among newborns whose mothers were compliant with acupuncture should be confirmed in future studies. This clinically relevant difference, if confirmed, could result in earlier discharge for mother-infant dyads. Investigators working with similar populations would be well advised to consider a “run-in” period to measure compliance prior to randomization. As well, our power to detect differences in study outcomes was further limited by the fact that our sample size calculations were based on longer periods of morphine treatment than those observed in our study.

We conducted our study in a hospital unit designed specifically for chemically dependent women. As such, women readily disclosed the nature of their drug dependency without fear of retribution or random urine testing for themselves or their newborns. There is no coercion to participate in the study as the unit is funded through our provincial health care program and is therefore free of charge. However, the variety of services available to women on the ward, such as Narcotics Anonymous meetings, yoga, and other activities, may have reduced the potency of the acupuncture intervention.

Controversy regarding the relationship between methadone dose and the incidence and severity of (NAS) [23] raises new challenges for evaluating the effectiveness of measures aimed at reducing maternal substance use. It also begs the question of whether or not there are more subtle measures of opioid sequelae in the newborn that could be measured or whether long term sequelae may exist in the absence of NAS. Some authors have noted that opioid exposed infants may exhibit subacute NAS symptoms for weeks to months after birth [55]. If the severity of NAS remains problematic as a measure of maternal drug exposure, then ongoing studies will benefit from more accurate measures of maternal substance use that would not in turn deter women from participation in research studies. Ideally such studies would be conducted in an outpatient setting in which assessment of maternal exposure could include self report, voluntary testing, and observation by outreach/case workers and counsellors.

## Conclusions

Our findings, while not conclusive, should encourage the continuing study of acupuncture as a safe and “low tech” intervention which can be administered during pregnancy. Our findings of potentially shorter duration of NAS among newborns of mothers who received acupuncture, while limited to a small group, deserve further study. Potential investigators should be aware that while chemically dependent women are willing to participate in a randomized controlled trial of acupuncture, attrition rates are high and measures should be taken to enrol women who have demonstrated the ability to remain compliant with acupuncture protocols.

## Abbreviation

NAS: Neonatal Abstinence Syndrome.

## Competing interests

The authors declare that they have no competing interests.

## Authors’ contributions

PJ, LD, AK, PT, and RA contributed to the conception and design of the study. PJ, LD, and AK, contributed to the analysis and interpretation of data. All authors undertook critical revision of the manuscript for intellectual content and approved the final version of the manuscript submitted for publication.

## Authors’ information

PJ is a registered nurse and perinatal epidemiologist whose research focus is aimed at birth outcomes among women from marginalized populations. LD is a practicing doctor of traditional Chinese medicine. AK is a perinatal epidemiologist whose research focus is addiction in pregnancy. PT is a pediatrician. RA is a family practice physician and Medical Director, Perinatal Addictions, BC Women’s Hospital in Vancouver, British Columbia, Canada.
